# Evaluation of’the Buddy Study’, a peer support program for second victims in healthcare: a survey in two Danish hospital departments

**DOI:** 10.1186/s12913-022-07973-9

**Published:** 2022-04-27

**Authors:** Katja Schrøder, Tine Bovil, Jan Stener Jørgensen, Charlotte Abrahamsen

**Affiliations:** 1 Department of Public Health, Research Unit of User Perspectives, University of Southern Denmark, Odense, Denmark; 2 Department of Clinical Research, Research Unit of Gynaecology and Obstetrics, Odense University Hospital, University of Southern Denmark, J.B. Winsløws Vej 9, 5000 Odense C, Denmark; 3 Department of Public Health, Research Unit for Epidemiology, Biostatistics and Biodemography, University of Southern Denmark, Odense, Denmark; 4 Department of Regional Health Research, University of Southern Denmark, Odense, Denmark

**Keywords:** Adverse events, Emergency medicine, Healthcare providers, Midwifery, Obstetrics, Peer support, Second victims, Support programs

## Abstract

**Background:**

Healthcare professionals involved in adverse events may suffer severe physical and emotional distress in the aftermath. Adequate support is critical to an overall culture of safety for any healthcare institution. This study evaluates a formalised peer support program, ‘the Buddy Study’, in two Danish university hospital departments. The program consists of a 2-h seminar about second victims and self-selected buddies to provide peer support after adverse events.

**Methods:**

The study design involved a cross-sectional survey comprised of two close-ended questionnaires evaluating the Buddy Study seminar (Q1) and the Buddy Study program (Q2), along with two open-ended questions and three individual interviews for more elaborate answers.

**Results:**

Out of the 250 HCPs employed in both departments, 191 midwives, physicians, and nursing assistants completed Q1 and 156 completed Q2. The seminars were evaluated positively; 91.6% were satisfied with the overall content of the seminar, and 69.1% agreed that insight into how other people may react to adverse events has helped them contain their own reactions or emotions. Assessments of having the Buddy Study program in the department or using or being used as a buddy were more diverse, yet overall positive. Three benefits of the program were identified: the program i) has encouraged an open and compassionate culture; ii) has caused attentiveness to the wellbeing of colleagues; and iii) the self-selected buddy relationship has created a safe space for sharing. Additionally, three challenges or shortcomings were identified: i) although peer support is valuable, it should not stand alone; ii) informal peer support is already in place, hence making a formalised system redundant; and iii) the buddy system requires continuous maintenance and visibility.

**Conclusions:**

The overall evaluation of the Buddy Study program was positive, suggesting that this type of formalised peer support may contribute to a rapid and accessible second-victim support program in healthcare institutions. A key principle for the Buddy Study program is that relationships are crucial, and all buddy relationships are based on self-selection. This seems to offer a safe space for health care professionals to share emotional vulnerability and professional insecurity after an adverse event.

## Background

Multiple studies have demonstrated the impact of adverse events on healthcare professionals (HCPs), and the term ‘second victim’, coined by Wu in 2000 [1], is now considered a highly relevant phenomenon for all healthcare institutions [2, 3]. Second victims are HCPs involved in an unanticipated adverse patient event, in a medical error and/or a patient-related injury and become victimized in the sense that they are traumatised by the event [4]. They may experience psychological and physical distress, fear, loss of self-esteem, guilt, anger, frustration, fear of continued practice, and even post-traumatic stress disorder in the aftermath of the event [2, 5, 6].

While patient safety efforts target preventative measures to reduce adverse events, the management of their aftermath seems to be trailing behind, especially regarding support for the HCPs involved. This lack of support may impair their health, reduce job satisfaction, and compromise their ability to provide safe, compassionate, and high-quality health care [7, 8]. A recent systematic review concluded that only a few second-victim support interventions are instituted worldwide, and the authors recommend that healthcare institutions prioritise support structures for HCPs facing adverse events [9]. In total, 12 second-victim support programs were implemented between 2006 and 2017. The common goal for all programs was to identify and reduce second victims’ emotional distress, foster HCPs’ coping strategies, and promote individual resilience. Beneficial effects were identified for workplace safety and support culture in general and the affected staff as well as the peer responders. However, because only a few studies have provided preliminary data on the beneficial effects of the support programs, the authors of the review were unable to provide a synthesis of the programs’ effectiveness [9].

Several reasons may explain this lack of sufficient empirical data. Firstly, this is still a young research field, and the development, implementation, and evaluation of support programs require several years. Consequently, many papers have only aimed to describe the development and the implementation of the programs [9]. Secondly, the confidential nature of adverse events and peer support encounters makes data collection challenging [9]. An inherent conflict exists between assuring confidentiality for second victims and evaluating the outcome of the encounters [10]. Thirdly, sufficient methods have not been established to measure the performance or effectiveness of second-victim support programs.

All these reasons were considered when ‘The Buddy Study’; a new peer support program for second victims was developed and implemented in two Danish hospital departments. The aim of this study is to evaluate the Buddy Study program by assessing HCPs’ experiences with having the program in the department, attending the compulsory seminar, and using a buddy or being activated as a buddy.

## Methods

### Study design

The study was designed as a cross-sectional questionnaire survey. In addition, three individual interviews with participants were conducted to include more elaborate answers in the evaluation.

### The Buddy Study program

The objective of the Buddy Study program is to facilitate peer support after adverse or traumatic events through a formalised buddy system and a compulsory seminar about second victims and peer support. Adverse events were defined as patient events with unanticipated adverse outcomes, medical error and/or patient related injuries. Traumatic events could include situations not associated with safety incidents, such as patient death or workplace violence.

On the basis of literature searches, our previous research, and five focus groups with a total of 21 midwives and physicians [11], the research team defined the following five underlying principles for the peer support program. *i) Recognition of exposure to adverse or traumatic events as a fundamental condition for HCPs.* In modern healthcare, the prevention of adverse events is pivotal. However, the management of the aftermath of these events is also crucial, and HCPs should be acknowledged for working in complex systems with the risk of involvement in severe adverse or traumatic events. *ii) Organisational responsibility towards all employees every time.* Focus groups revealed inconsistency in the support offered after adverse events, even within the same department. Second-victim support resources should be reliable for all HCPs and not based on inconsistent or random assessments of when support or debriefings should be facilitated. *iii) Relationships are of central importance.* Second victims may feel guilt, fear, and loss of self-esteem, which are emotions one may prefer to disclose only in an already established and safe relationship. In recognition of the importance of a personal relationship, HCPs should be able to select a peer supporter of their own choice. *iv) Build on existing resources in the departments.* Although management may have the will to invest in the safety and health of the employees, the general soaring healthcare budget may only allow minor additional costs. HCPs are all trained to care for people in crisis, and studies have shown that colleagues are the preferred resource of support for second victims. They are a valuable resource for a support program. *v) Research-based evaluation of the intervention.* The program should be designed as a research intervention to contribute to the body of evidence on the effectiveness of second-victim support resources.

Grounded in these underlying principles, the Buddy Study was designed to encompass 1) a compulsory seminar, 2) a self-selection of two Buddies, and 3) a system for buddy activation and response after adverse events.

The purpose of the compulsory 2-h seminar was to provide participants with knowledge about the second victim phenomenon and stress responses that may be associated with a traumatic or adverse event. The seminars were conducted for smaller groups of 5–15 participants at the time. Through interactive exercises, participants shared their experiences and became aware of support needs after a traumatic or adverse event. Furthermore, participants were informed about the buddy system program, how to activate one’s buddy, how to respond as someone’s buddy, and when to seek further support from management or a psychologist. The seminar addressed all participants as both potential second victims and buddies. The seminar was compulsory in order to communicate that adverse events are a general risk for all (junior as well as senior staff) and promote a shared understanding of second victims and peer support.

At the end of the seminar, all participants named two colleagues whom they would want to be their buddies in case of an adverse event (on a note in a closed envelope). The selected colleagues were then asked to consent to be that person’s buddy. Eventually, a list with names of all HCPs and names of their two buddies was created, and the support program was ready for implementation.

In case of an adverse or traumatic event, the buddy system can be activated through the involved HCP, a colleague, or the manager. Although it was compulsory to participate in the seminar and to select two buddies, using a buddy after an adverse event was voluntary. If the involved HCP provided consent, the manager or charge midwife would contact their buddy. No information about the event would be passed on, nor any assessment of the emotional state of the HCP. The only information provided would state that ‘NN has been involved in an adverse event and he/she wants to activate his/her buddy’. Subsequently, it was the responsibility of the buddy to reach out and contact the HCP, preferably within the first 24 h. The time frame for a buddy contact was 2 h, which could be spent in any way the HCP and the buddy agreed on—phone calls or encounters in or out of the workplace, several shorter sessions, or fewer longer sessions—over a 4-week period. In a previous study, less than half of the respondents agreed to a great or some extent that their *colleagues provided meaningful and sustained support after the event* [12]. Consequently, a four-week time period was chosen to ensure *sustained* support for the involved HCP balanced with the potential risk of burdening the buddy over time. After four weeks, other measures should be offered, sooner if needed. The only stipulation was that the buddy encounters would happen outside working hours to ensure a space for private, unguarded conversations. The buddy would be paid for the 2 h of work.

The Buddy Study program was implemented, and the evaluation was conducted 18 months after the last seminar.

### Respondents and data collection—questionnaire survey

HCPs from two departments at Odense University Hospital in Denmark participated in the Buddy Study program: all midwives in the Department of Obstetrics and Gynaecology (OB-GYN) in Odense and Svendborg and all physicians at the Internal Medicine and Emergency Department (IME) in Svendborg. Both departments are involved in high degrees of acute patient care and decision-making.

The first questionnaire (Q1) evaluating the Buddy Study seminars was handed out to all participants at the seminars between May 2018 and April 2019. The seminar programme included time to complete and return the questionnaire. A second questionnaire (Q2) evaluating the Buddy Study program was handed out at general staff meetings in October and November 2020. Because of COVID-19 restrictions, only small groups could attend these meetings, and questionnaires were subsequently distributed via e-mail invitation with a survey link to increase the response rate. The survey was created using REDCap (Research Electronic Data Capture), and all paper versions were entered into the same database in REDCap.

Both departments have a high turnover of staff because of temporary employment during training for resident doctors, maternity leave, etc. Therefore, the study population changed during the 18 months and was not identical for Q1 and Q2.

During the study period, the use of the buddy system was registered, including the type of event that activated the need to use one’s buddy.

### Questionnaire

To assess the participants’ experiences with the Buddy Study program, 22 close-ended items were created. The first questionnaire (Q1) included nine items addressing satisfaction with the seminar, learning outcomes, sense of safety, and attitudes towards the buddy program. In the second questionnaire (Q2), five items evaluated the Buddy Study program, addressing the overall experience with the program—including the seminar and the derived change in supportive culture, awareness, and sense of security. Furthermore, eight items specifically addressed the experiences of those who had either used a buddy or been activated as a buddy.

All 22 items were specifically constructed to evaluate the Buddy Study program and were face validated by three HCPs, resulting in only minor adjustments. All items were composed of a five-point Likert scale ranging from ‘strongly disagree’ to ‘strongly agree’.

Furthermore, the survey included two open-ended questions: 1) ‘In your own words, how did you experience using your buddy?’ and 2) ‘In your own words, how did you experience being a buddy for a colleague?’. An additional box for free-text comments was available at the end of the survey.

Finally, both questionnaires (Q1 and Q2) contained three categorical items on gender, professional background, and seniority, and Q2 included three additional items regarding the length of employment in the department, attendance at the Buddy Study seminar (yes/no), and whether two buddies were chosen (yes/no). Within a time period of 18 months, there is a turnover of staff, and items on seminar attendance and selection of buddies were included to keep track on the level of implementation of the Buddy Study program.

### Respondents and data collection—interviews

Upon the completion of Q2, an e-mail invitation to participate in an individual online interview was distributed to all participants in both departments. Because of COVID-19 restrictions, interviews were conducted and recorded via Zoom. A semi-structured interview guide based on preliminary results from the survey was developed, which focused on i) experiences with having a buddy program in the department, ii) peer support in general, iii) the meaning of self-selected relations, and iv) shortcomings of the program. Interviews were conducted by the first author (KS).

### Data analyses

Questionnaires with three or more missing responses were excluded, and the remaining missing responses were omitted from the final analyses. Respondents’ characteristics are expressed as proportions. To compare the two groups of respondents, chi-square tests were performed. The response options of the nine items assessing the participants’ experiences with the seminar, four items on using a buddy, and four items on being activated as a buddy were collapsed into three categories: ‘agree’ (agree/strongly agree), ‘neither agree nor disagree’, and ‘disagree’ (disagree/strongly disagree). The results are expressed as proportions. The five items evaluating the buddy program in general in Q2 were collapsed into’agree’ (to a large extent/ to some extent), ‘neither agree nor disagree’ (do not know), and ‘disagree’ (to a lesser extent/not at all). The results are expressed as proportions. Data analyses were performed using STATA 16.0 software (StataCorp 2019/College Station, TX, USA).

Each event leading to a Buddy contact was registered by type and number.

When performing qualitative data analysis, audio recordings of the interviews were listened to several times, and selected excerpts were transcribed, as proposed by Miles et al. [13]. The transcribed data were pooled with the data from the open-ended questions and free-text comments in the survey. Rooted in an essentialist paradigm, and in line with the descriptive, thematic analyses of the survey data, an essentialist approach was followed for the analysis of the qualitative data assuming a simple, unidirectional relationship between meaning and experience and language, as proposed by Braun and Clarke [14]. This entailed reporting experiences, meanings and the reality of participants, and performing a descriptive content analysis to comprehensively summarise the data by staying close to the words and events. This is an adoption of Sandelowki’s exposition of qualitative descriptive studies, representing language as a ‘vehicle of communication’, not itself an interpretive structure that must be read [15]. The coding process departed from the pre-existing categories in the survey and the interview guide. The codes were modified by the first author (KS) during the next cycles of coding, and interviews were listened to again to select further excerpts for transcription. Descriptive themes were selected and placed in a matrix with condensed descriptions and illustrative quotes.

## Results

A total of 250 HCPs (all physicians in IME, and all midwives and nursing assistants in OB-GYN) were employed in the two departments, of which 198 participated in the seminars, where all attendees responded to Q1 (response rate 79% of the entire population/100% of the attendees). Seven questionnaires were excluded because of missing responses, leaving 191 for data analyses. After 18 months, 167 responded to Q2 (response rate of 67%). Because of missing responses, 11 questionnaires were excluded, leaving 156 for data analysis. Of the 156, 106 (67.9%) replied that they had participated in and evaluated the Buddy Study seminar, and 120 (76.9%) replied that they had chosen two buddies.

In total, 26 HCPs used their buddy, and 32 were activated as a buddy. Eight out of ten respondents were women, and more than 95% were midwives or physicians (Table 1).

**Table 1:** Characteristics of respondents evaluating the Buddy seminar and Buddy program *n* = 191 (Q1), *n* = 156 (Q2), (%)

Characteristics	Q1 *n* = 191, (%)	Q2 *n* = 156, (%)	*p*
**Sex**			
Female	149 (78.0)	124 (79.5)	0.248
Male	42 (21.9)	30 (19.2)	
Non-binary	-	2 (1.3)	
**Professional background**			
Midwife	91 (47.6)	86 (55.1)	0.146
Physician	92 (48.2)	68 (43.6)	
Nursing assistants	8 (4.2)	2 (1.3)	
**Seniority**			
0–4 years	68 (35.6)	44 (28.2)	0.005
5–9 years	49 (25.7)	25 (16.0)	
> 10 years	72 (37.7)	87 (55.8)	
Missing	2 (1.0)	-	
**Length of employment in this department**			
0–1 years	-	35 (22.4)	
1–3 years	-	26 (16.7)	
> 3 years	-	95 (60.9)	
**Attendance ‘The Buddy Study seminar’?**			
Yes	-	106 (67.9)	
No, I was not offered	-	31 (19.9)	
No, I could not attend	-	19 (12.2)	
**Chosen two buddies?**			
Yes	-	120 (76.9)	
No, I was not offered	-	16 (10.3)	
No, I chose not to participate	-	9 (5.8)	
I do not remember	-	11 (7.0)	

### Evaluation of the Buddy Study seminar (nine items)

The Buddy Study seminar was evaluated positively (Table 2). Most participants (92.7%) believed that compulsory participation in the seminar provided mutual insight and understanding (item 4), but 8.4% would have preferred the ability to choose whether to participate (item 8). The majority (87.4%) felt prepared to become a buddy for their colleagues (item 2), and 90.6% felt positively about testing the buddy program in the department (item 9).

**Table 2:** Evaluation of The Buddy Study seminar, *n* = 191 (%)

Item	Agree	Neither agree nor disagree	Disagree
1. I have gained knowledge about the second victim phenomenon	188 (98.4)	2 (1.0)	1 (0.5)
2. I feel prepared to become a buddy for my colleagues	167 (87.4)	22 (11.5)	2 (1.1)
3. I am satisfied with the overall content of the seminar	175 (91.6)	13 (6.8)	3 (1.6)
4. Compulsory participation in the seminar gives a mutual insight and understanding	177 (92.7)	12 (6.3)	2 (1.0)
5. Insight into how other people may react to adverse events has helped me contain my own reactions or emotions (*n* = 190)	132 (69.1)	55 (28.8)	3 (1.6)
6. The possibility to talk to a buddy has provided me with a sense of safety	135 (70.7)	48 (25.1)	8 (4.2)
7. I fear that being a buddy for someone else will be a burden to me (*n* = 189) ^a^	18 (9.4)	25 (13.1)	146 (77.4)
8. It annoyed me that the seminar was compulsory – I would prefer to choose whether to participate myself (*n* = 189) ^a^	16 (8.4)	49 (25.7)	124 (64.9)
9. Right now, I feel positive about testing the Buddy program in our department	173 (90.6)	16 (8.4)	2 (1.0)

^a^reverse worded compared to the rest items

### Evaluation of The Buddy Study program in the department (five items)

Overall, the Buddy Study program was evaluated positively, yet more diverse than the evaluation of the seminar. The majority replied that the program had increased attentiveness to one another and sense of feeling safe. But only a third felt that the program had led to more openness after adverse events (Table 3).

**Table 3:** Evaluation of the Buddy Study program in the department, *n* = 156 (%)

Item	Agree	Neither agree nor disagree	Disagree
10. Having a Buddy program in our department made me feel safe (*n* = 154)	91 (58.3)	25 (16.0)	38 (24.4)
11. I have experienced that the Buddy program has encouraged more attentiveness to one another after adverse events (*n* = 155)	82 (52.6)	36 (23.1)	37 (23.7)
12. I have experienced that the seminars and the Buddy program have contributed to more inter-collegial talks about how adverse events may affect us (*n* = 154)	57 (36.5)	49 (31.4)	48 (30.8)
13. The Buddy program has made me more open to my colleagues about how I may feel in the aftermath of adverse events	65 (41.7)	24 (15.4)	67 (42.9)
14. The Buddy program has made me more open to my manager about how I may feel in the aftermath of adverse events (*n* = 152)	53 (34.0)	27 (17.3)	72 (46.1)

### Evaluation of using a buddy or being activated as a buddy (8 items)

Of the 156 respondents, 26 used one of their buddies during the study period. Of the 26 respondents, 34.6% had seniority of 0–4 years, 15.4% had seniority of 5–9 years, and 50.0% had seniority of 10 years or more (data not shown in table).

Positive experiences with using one’s buddy ranged from 65.4% to 80.8%, whereas the proportion of those who disagreed that the experience was positive ranged from 11.5% to 15.4%, and the proportion of those who neither agreed nor disagreed ranged between 7.7% and 19.2% (Table 4).

**Table 4:** Questions if you have used your buddy *n* = 26 (%)

	**Agree**	**Neither agree nor disagree**	**Disagree**
15. It was a help that somebody reached out for me	19 (73.1)	3 (11.5)	4 (15.4)
16. Talking to my buddy has made me feel less alone with my experiences	21 (80.8)	2 (7.7)	3 (11.5)
17. My buddy has provided a room for professional reflection after the event [on obstetrical or midwifery matters]	17 (65.4)	5 (19.2)	4 (15.4)
18. My buddy has given me emotional support after the event	20 (77.0)	3 (11.5)	3 (11.5)

Furthermore, 32 HCPs were activated as buddies for a colleague during the study period. In total, 21.9% HCPs had seniority of 0–4 years, 18.8% had seniority of 5–9 years, and 59.4% had seniority of 10 years or more when activated as a buddy (data not shown in table).

Positive experiences (‘agree’) with being activated as a buddy ranged from 68.8% to 81.3%, negative experiences (‘disagree’) ranged from 3.1% to 9.3%, and the proportion of those who neither agreed nor disagreed ranged from 15.6% to 25.0% (Table 5).

**Table 5:** Questions if you have been activated as a buddy *n* = 32 (%)

	**Agree**	**Neither agree nor disagree**	**Disagree**
19. I have felt prepared to take on the role as a buddy for my colleagues	23 (71.9)	6 (18.8)	3 (9.3)
20. I felt it was a burden when I had to be a buddy for my colleague^a^	2 (6.2)	6 (18.8)	24 (75.0)
21. I experienced that I was able to help my colleague as a buddy	26 (81.3)	5 (15.6)	1 (3.1)
22. Being a buddy for my colleague has been an opportunity to reflect upon my own experiences with adverse events and my reactions to those	22 (68.8)	8 (25.0)	2 (6.2)

^a^reverse worded compared to the rest items

### Registrations

During the 18-month study period, 29 buddy calls were registered. The types of events causing the activation of a buddy were death (including foetal or neonatal death) (*n* = 12), cardiac arrest (*n* = 1), unexpected adverse outcomes (*n* = 4), medical error (= 1), complaints from patients (*n* = 2), violent or threatening behaviour from patients or relatives (*n* = 4) and other/don’t know (*n* = 5).

### Qualitative findings

From the individual interview data from three HCPs and 35 free-text comments or responses to the open-ended questions in Q2, we identified three benefits of the program: i) encouragement of an open and compassionate culture, ii) attentiveness to the wellbeing of colleagues, and iii) self-selected relationships create a safe space for sharing. Additionally, we identified three challenges or shortcomings of the program: i) peer support is valuable but should not stand alone, ii) informal peer support is already in place, and iii) the Buddy system requires continuous updating and visibility. Table 6 provides further condensed descriptions and illustrative quotes for each of the benefits and challenges or shortcomings.

**Table 6:** Summary of descriptive content analysis of qualitative data

	**Condensations of descriptions**	**Illustrative quotes**
Benefits of the program		
**Encouragement of an open and compassionate culture**	Contributes to an increased awareness about the implications of adverse events	” The seminar showed us that we should talk more about this. I think it has brought on a more open culture.”
	Enforces a sense of being part of a team, combats loneliness	“I feel like I am part of a team with this system.”
	Communicates openness to share difficult experiences and emotions. Legitimises feelings of vulnerability in the aftermath and encourages to reach out	“My buddy allowed me to cry and tell everything without any judgement. She cried with me.” “Without my buddy I would never have been able to go to sleep those first couple of days.”
**Attentiveness to the wellbeing of colleagues**	Knowing that someone will reach out to you in case of an adverse event provides a sense of safety	“One thing is talking about it there and then. But knowing that someone reaches out to you the following days makes me feel safer.”
	Establishes an awareness that it can happen to all. Greater willingness to talk about errors in general	” We pay more attention to each other now.”
	Rewarding to be able to help a colleague (as a buddy). May give reason to reflect on (and heal) own previous experiences with an adverse event	“I have activated buddies for someone else. And their reactions are like “Yes! Of course!”. They really want to help and support their colleagues.”
**Self-selected relationships create a safe space for sharing**	It is easier to share emotional distress with someone you know and trust	“My buddy is a junior like myself and has been incredibly supportive. She can relate to my experiences.” “Having a buddy is of great value to me.”
	Evaluation of clinical decisions happens in a safe space. This facilitates learning without triggering defensive responses	“Going through the event and my clinical decisions with someone I trusted was… I learned a lot.”
Challenges or shortcomings of the program		
**Peer support is valuable but should not stand alone**	Support from management is crucial	“Difficult to be someone’s buddy when there is no support from our manager. I felt a bit powerless in my effort to help.” “My manager is very supportive in these cases. It is so important to have a manager like that.” “You need the acknowledgement from your manager. Something like ‘I know you a going through a rough time. I see you’.”
	Organisational follow-up can still be deficient, even with a buddy system	“A debriefing, that’s it. And in some cases, that is not enough.” “You may be too exhausted to reach out yourself.”
**Informal peer support is already in place**	A formalised system is unnecessary, especially in smaller units	“I work in a smaller team, where I feel acknowledged and listened to, when things are difficult.” “We have a very compassionate culture in our unit, so the buddy system seems unnecessary here.”
**The Buddy system requires continuous updating and visibility**	The turnover of staff in large departments is a challenge	“The project was not visible enough. I was not included when I started at the department.”
	It was unclear when to activate the buddy system	“I have considered activating my buddy. But I was uncertain whether the event was severe enough. So, I didn’t.”

## Discussion

Most participants evaluated the Buddy Study seminar positively, whereas the evaluation of the Buddy Study program in the department was more diverse, with only 37–58% of participants agreeing on positive experiences of the program. Assessments of either using or being used as a Buddy were positive overall. Three benefits and three challenges or shortcomings of the program were identified.

The Buddy Study program is similar to other support programs (such as the forYOU Team, RISE, or YOU Matter Program [10, 16, 17], but differs on two central aspects: i) self-selected relations within the department instead of specialised training for a small team of hospital-wide support team, and ii) peer supporters (buddies) receive financial remuneration equivalent of two hours wages. The rationale behind this monetary compensation is to emphasise that providing support systems for second victims is the responsibility of the employer, not the employees. In a new field, we believe that it is of paramount importance to design and test different types of interventions to gain knowledge about the benefits or shortcomings of the different approaches. The discussion of the findings follows the structure of the five underlying principles for the Buddy Study program described earlier.

The compulsory seminar and the selection of two buddies complied with the first principle: the recognition of exposure to adverse events as a fundamental condition for all HCPs. A voluntary approach could have suggested that only some HCPs require support in the aftermath of an adverse event and further reinforced a culture in which seeking support is considered a sign of weakness. With this project, we aimed to achieve the exact opposite: a culture in which adverse events are acknowledged as a fundamental condition in healthcare and support is a natural part of the aftermath. Moreover, our data oppose the general belief that only junior staff are affected by the emotional stress of adverse events. In our study, HCPs with both low and high seniority used their buddies. A compulsory seminar for all was assumed to support mutual insight and understanding of second victims and contribute to a more open and supportive culture. This assumption was generally confirmed, as the majority agreed that compulsory participation led to mutual understanding and that insight into others’ reactions to adverse events helped them contain their own reactions and emotions. Furthermore, over a third of the participants believed that the seminar contributed to more inter-collegial talks about the impact of adverse events. However, although it was compulsory, 23% did not choose two buddies during the study period, because they were not offered this choice (10%), they chose not to participate (5%), or they did not remember the reason (7%). This indicates that the program did not achieve full implementation and possibly, that HCPs commencing at the department during the study period were not introduced to the program.

Although participation in the seminar and choosing two buddies was compulsory, using a buddy after an adverse event was voluntary. The underlying reason for this distinction was a comprehensive understanding of the diversity in reactions and supporting the needs of the individual HCPs. Although the organisation has a responsibility towards all employees every time (the second principle), this does not imply a normative approach to the support needs of the individual. Hence, a buddy could only be activated when the HCP had provided consent. Furthermore, adverse events have a broad range of content and the consequences of, and support needs may vary according to the severity or the frequency of the events. This may explain why only 77% agreed that they their buddy had given them emotional support after the event—some events may call for professional advice and evaluation of the cause of events rather than emotional support.

After 18 months, 26 HCPs had activated their buddy (out of 29 registered buddy calls) and evaluated that experience very positively. Other support programs reported that smaller numbers use the programs than anticipated and justified this by the limited awareness of the program or fear of blame and reluctance to show vulnerability [9]. In the present study, the interviewees did not mention fear of blame. It is worth considering whether a certain number of buddy calls should be considered indicative of the success of the program. It seems that the Buddy Study program has contributed to a more compassionate work culture with an increased focus on the impact of adverse events among colleagues, which may reduce the use of a formalised system.

The third principle, that relations are of central importance, was emphasised during the interviews. Self-selected relations were considered to add a greater sense of safety and to encourage a general sense of responsibility toward each other. Moreover, talking to a peer with the same background and training was considered to provide more qualified professional assessment of clinical decision-making than calling a hotline would be able to provide. Similarly, a Dutch interview study found that most interviewees were sceptical about a hospital-wide solution because this was considered to emphasise the presence of a cultural problem with the acceptance of vulnerability and support needs. They found that physicians should ideally receive support from a direct peer who could help them cope with their emotions and reflect on their professional performance. Contrary to recent developments in healthcare organisations, participants did not request a hospital-wide support team [18]. Concerns about whether buddies in this programme have adequate training for the task could be raised. Other programs have required training, attendance at meetings, simulation exercises and/or post-encounter debriefings for all peer responders [10, 17]. However, in this program we consider HCPs to be qualified to provide support for human beings in crisis, and this is their core competence as buddies providing psychological first aid. Because 37% to 58% of the responders agreed to the items evaluating the Buddy Study program in general, it seems that a fair proportion of the HCPs experienced benefits from the program (Table 3). However, it should be noted that between 24 and 43% did not agree with these items, meaning that they did not experience the positive effects from the program. This is a key observation and could be interpreted as an expression of one or all three of the identified challenges or shortcomings of the program: peer support should not stand alone, informal peer support is already in place for some (hence the Buddy program seems redundant to them), and the Buddy system requires continuous updating and visibility. The failure of the latter will inevitably lead to a lack of awareness and use of the program. It is also important to remember that one size does not fit all, and follow-up practices should accommodate different, individual needs in the workforce.

The positive evaluations of being activated as someone’s buddy indicate an unexploited potential to build on existing resources in the departments (the fourth principle). Placing the support program within the department may ensure long-term support and awareness, firstly because the buddies will remain co-workers long after the event, and secondly because the program involves the entire group of co-workers in the department, which potentially sustains a supportive and compassionate culture.

### Strengths and limitations

The fifth and final principle of the programme was to conduct a research-based evaluation of the intervention. Therefore, we developed and face-validated a questionnaire for this specific purpose. The content of all questions was found to be relevant, comprehensible, and comprehensive after minor adjustments. However, the questionnaire was not psychometric tested. A risk of HCPs over-reporting what was socially acceptable to others was reduced as both questionnaires were completed anonymously. Furthermore, our results indicate a variety of responses.

Due to the pressure of Covid-19 in the clinical departments, we only managed to recruit three HCPs for the qualitative interviews.

An essential aspect of the evaluation is to consider to which level the intervention was implemented. Almost 30% of all employees in the two departments did not attend the seminar, which means that they were not fully informed, and the intervention was not fully implemented. The response rate for Q1 was 100% of the attendees at the seminars, and for Q2 it was 67%. Unfortunately, we are unable to account for non-attender and non-responder characteristics and are thus unable to account for potential non-response bias. During the study period, the use of the Buddy system was registered manually in a folder. Since 32 participants to Q2 responded that they had been activated as a buddy, and only 29 registrations were found, it seems that the registration is inadequate or that a few activated both buddies. These limitations illustrate the challenges of conducting interventional research in a busy clinical setting on a subject that is not possible to protocolise as a clinical trial. Consequently, the findings may have been influenced by external factors not accounted for in the scope of our study.

The intervention was performed in two departments at a single institution, included only physicians, midwives and nurse assistants and the majority of participants were women. This makes it difficult to generalise the findings to other institutions. However, we believe that the results are relevant for other healthcare institutions aiming to improve their support of second victims. Both departments decided to continue with the Buddy program after the research period since staff and management considered it to be an important and useful support tool to handle the aftermath of adverse events.

## Conclusion

The Buddy Study program provides peer support to HCPs after adverse events. It is built on the pre-existing resources in the departments, and it is considered a strength that all Buddy relations are self-selected, which creates a safe space for disclosing emotional vulnerability and professional insecurity.

The compulsory 2-h seminar was evaluated positively, particularly because it allowed participants to gain knowledge about the second victim phenomenon and obtain mutual insight and understanding with colleagues. Some of the participants reported that having the Buddy Study program in the department made them feel safe, that it encouraged attentiveness to one another after adverse events, and that it encouraged an open and compassionate culture. Using a buddy after adverse events was found to be helpful, and most of the respondents found both professional and emotional support with their buddy. Being activated as a buddy for a colleague was found to be rewarding both in terms of being able to help someone else and as an opportunity to reflect upon one’s own experiences.

However, the formalised peer support program should not stand alone. Management plays a significant role in handling the aftermath of an adverse event. For those who already had well-established relationships and a compassionate culture in their team, the formalised buddy program seemed less relevant.

## Data Availability

The data that support the findings of this study are available from the corresponding author upon reasonable request and after obtaining adequate permission according to Danish law.
